# Organic photovoltaic prediction model based on Bayesian optimization and explainable AI

**DOI:** 10.1038/s41598-025-18632-4

**Published:** 2025-09-16

**Authors:** Sara Abdelghafar, Heba Alshater, Lobna M. Abouelmagd, Ashraf Darwish, Aboul Ella Hassanien

**Affiliations:** 1 School of Computer Science, Canadian International College (CIC), Cairo, Egypt; 2 Department of Forensic Medicine and Clinical Toxicology, Menoufia University Hospital, Shebin El-Kom, Egypt; 3 Computer Science Department, Misr Higher Institute for Commerce and Computers, Mansoura, Egypt; 4 College of Information Technology, Ahlia University, Manama, Kingdom of Bahrain; 5 Faculty of Computers and Artificial Intelligence, Cairo University, Cairo, Egypt

**Keywords:** Bayesian optimization, Bootstrap aggregating, Explainable artificial intelligence, Intelligent chemistry, Machine learning, Organic photovoltaics, Multi-objective predictive model, Renewable and sustainable energy, Photovoltaics, Computational science, Solar cells

## Abstract

Over the decades, as industrialization progressed, energy has been a critical topic for scientists and engineers. Particularly, photovoltaic technology has drawn great attention in the renewable energy industry as an environmentally clean technology for converting sunlight into electricity. However, the complexity of energy chemistry and the need for novel materials to improve solar cell efficiency and cost-effectiveness have led to challenges in establishing rules beyond empirical observations. Machine learning models are being developed to streamline the prediction process and efficiently predict photovoltaic parameters. This paper proposes a novel hybrid-optimized multi-objective predictive model to predict the photovoltaic parameters: open-circuit voltage (Voc), current density (Jsc), fill factor (FF), and power conversion efficiency (PCE). The proposed model is based on Bayesian Optimization (BO) with the ensemble Bootstrap Aggregating (Bagging) decision tree. The proposed model integrates with the Explainable Artificial Intelligence (XAI) using the SHAP (Shapley Additive Explanations) values to introduce feature importance analysis that provides valuable insights into the impact of individual features on prediction outputs. The proposed model, named BO-Bagging, achieves high prediction accuracy, with an average high correlation coefficient of r = 0.92, a coefficient of determination of R^2^ = 0.82, and a Mean Square Error (MSE) of 0.00172. In terms of complexity, the BO-Bagging model has a short processing time that is indicated with an average training time of 182.7 s and an average inference time averaging 0.00062 s. Also, the number of predicted observations per second is measured by prediction speed, which results in good prediction accuracy with an average of 2188.4 and model size with an average of 10,740.4 KB. Finally, the proposed model’s primary critical operations across each phase, from training to predicting the final outputs, are represented by 108 floating-point operations per second (FLOPS). All of these results demonstrate the proposed model’s accuracy and high efficiency in intelligent chemical applications.

## Introduction

The demand for sustainable energy solutions is rising along with the world’s environmental challenges. One of the most promising solutions to these challenges is solar energy, which is a clean and renewable energy source. A type of solar cell known as a photovoltaic cell is a renewable energy source that uses photovoltaic panels to turn sunlight into electricity. High-performance photovoltaic technologies can significantly increase global energy production and reduce environmental impacts^1^.

Photovoltaic cells are made of a semiconductor material that absorbs sunlight and emits electrons and are classified into two types: classic inorganic silicon cells and newer organic cells made of polymers or small molecules^2^. Organic photovoltaic (OPV) is an innovative method of turning solar energy into electrical energy. The cost and manufacturing procedure of conventional inorganic material-based solar cells could be significantly reduced by using inexpensive organic materials^3^. The advantages of OPV include semi-transparency, adaptability, and competitiveness. However, due to open-circuit voltage losses and a narrow absorption spectrum, their high-power conversion efficiency is lower than that of inorganic photovoltaic cells. Because organic photovoltaic are made of carbon-based materials, they have lower efficiency and are more sensitive to environmental factors than inorganic photovoltaic, which are mostly made of silicon, have higher stability and efficiency, and are less sensitive to environmental factors^4^. As a result, the development of cost-effective, efficient production methods, material selection, and optimization methodologies is critical for enhancing OPV efficiency. Experiments are time-consuming and resource-intensive, making it challenging to screen new material combinations and estimate OPV performance via experimental and trial-and-error methods^5^.

The screening of novel material combinations and the effective prediction of OPV performance are being revolutionized by machine learning (ML). Materials science research has made extensive use of machine learning approaches to ascertain the characteristics and capabilities of current materials or identify novel materials with desired features. ML is becoming more and more popular because of its potential to increase productivity and help find new materials, especially in OPV material discovery and property prediction. Various ML methods are utilized to predict the performance of organic photovoltaic cells, often screening potential materials for organic solar cells based on power conversion efficiency (PCE) using various target datasets. A machine learning algorithm can accurately and quickly evaluate candidate OPV materials to discover high-performance candidates. Current ML models have high accuracy for PCE prediction but require additional chemical properties and molecular structure inputs. This laborious pre-acquisition process hinders their applicability and suggests an ideal ML model requiring only molecular structural information^6^. Kim et al.^7^ propose a novel computational structure descriptor that divides molecular structures into functional group units (FGs). The molecular structure is converted into a short integer matrix by combining the indices.

This novel descriptor makes it simple for machine learning models to track FGs and give more weight to those that significantly affect solar performance. This new descriptor dataset has been published by the authors in the public access benchmark^8^. The benchmark dataset presents a novel structural feature-descriptor platform that enables the FGs to be computationally associated with more PV parameters, such as fill factor (FF), open-circuit voltage (Voc), short-circuit current density (Jsc), and PCE, obtained from the molecular structures of donor and acceptor materials. It creates a low-dimensional matrix by splitting the molecule structure into FGs and giving each one a positive integer.

This paper proposes a novel hybrid optimized prediction model, BO-Bagging, based on the ensemble Bootstrap Aggregating (Bagging) decision trees and Bayesian Optimization (BO), for predicting PV parameters (Voc, Jsc, FF, and PCE) from donor and acceptor material molecular structures. Improving photovoltaic performance in solar cells through enhanced Voc, Jsc, FF, and PCE shows potential for next-generation photovoltaic technology. As a result, chemical properties are effectively extracted from molecular structures, allowing predictive models to be built with only structural feature descriptors and no additional chemical characterization. The proposed model, BO-Bagging, is based on Bayesian Optimization and Bootstrap Aggregated Decision Trees. BO is used to optimize the learning hyperparameters (minimum leaf size, number of learners, and number of predictors) of the ensemble Bootstrap Aggregated Decision Trees, which can handle complicated and non-linear interactions among variables. Furthermore, the proposed model displays the impact relationship between the optimally selected predictors and each OPV parameter (Voc, Jsc, FF, and PCE) using the SHAP (Shapley Additive Explanations) values. SHAP is an increasingly common approach in Explainable Artificial Intelligence (XAI) that provides a consistent and objective explanation of how each variable affects the model’s prediction output.

The proposed model is compared to five ensemble machine learning models that have been effectively used previously in related work on this benchmark dataset and resulted in the prediction of new OPV material. These models include light gradient boosting machine (LGBM), gradient boosting decision trees (GBDT), extreme gradient boosting (XGBoost), random forest (RF), and adaptive boosting (AdaBoost). The proposed model, BO-Bagging, exceeds all of these ensemble models in terms of accuracy for all OPV parameter target responses (Voc, Jsc, FF, and PCE), demonstrating its efficacy and superiority. The experimental results demonstrate that the proposed model is highly efficient, with correlation coefficients of 0.85, 0.92, 0.73, and 0.87 for Voc, Jsc, FF, and PCE, respectively. Furthermore, the proposed model is a reliable and efficient prediction model due to its low processing time and high prediction speed. In addition, the model performs consistently across validation, indicating a high level of confidence in the outputs’ generalizability and consistency.

The main contributions of this paper can be summarized as follows:

1. The paper proposes a novel multi-objective predictive model named BO-Bagging that accurately predicts essential OPV parameters such as Voc, Jsc, FF, and PCE, which improves material composition and power conversion efficiency.
2. The proposed model combines the strengths of both optimization and ensemble learning techniques to improve prediction accuracy by fine-tuning learning parameters and ensemble the number of predictors.
3. The proposed model outperforms five other ensemble learning models with fewer errors and higher correlations between experimental and predicted values, with less complexity, demonstrating robustness and predictive accuracy.
4. The proposed model incorporates SHAP feature importance analysis for insights into individual feature impact on prediction outputs.

The remainder of the paper is structured as follows. “Related work” section introduces related work. “Basics and background” section provides background on OPV screening and the problem statement, followed by the proposed model’s construction methods, which include decision trees, the ensemble bagging method, Bayesian optimization, and the SHAP method. The proposed predictive model is presented in “The proposed organic photovoltaic prediction model” section. “Experimental analysis and results” section details the dataset, performance metrics used for evaluation, and presents the experimental results, highlighting the effectiveness of the proposed model. Finally, “Conclusion and future work” section introduces the conclusion and future work.

## Related work

Recently, there has been an increasing interest in predicting models for various photovoltaic challenges, such as opaque solar prediction, which evaluates the quantity of solar radiation that will reach the Earth’s surface and is critical for energy management and grid stability. In addition, to estimate solar irradiance under uncertain or low-visibility situations, energy yield forecasts for PV systems, defect detection and diagnostics in PV arrays, and predictive maintenance scheduling^9,10^. While these approaches work at the system or environmental level, different research focuses on predicting photovoltaic performance at the material level, specifically the stability and power conversion efficiency (PCE) of organic photovoltaic cells. Machine learning methods are increasingly being used to predict PCE based on experimental data and chemical structures, accelerating the search for high-efficiency OPV materials^11^.

Recent work on predicting OPV performance using ML has been widely used with high accuracy. However, the most recent studies are accompanied by quantitative structure–property relationships (QSPR), which require other chemical characteristics (e.g., energy band gap and charge-transfer state energy) as well as molecular structure as input. The strict pre-acquisition process limits the practicality of these models.

This is why a unique structural feature-descriptor platform originated in^7^, which divides the molecular structure into functional groups (FGs) and assigns positive integers to each. By adding the assigned numbers, the desired molecular structure is expressed as a low-dimensional matrix. The assigned integers are fed independently into the ML method, allowing each group in the molecular structure to be computationally tracked. This enables the algorithm to apply weights to important FGs associated with PV parameters, resulting in an efficient extraction of chemical characteristics from molecular structures. The authors also present ML models that were effectively implemented using only structural feature descriptors without any additional chemical characterization. The ML models were built using five ensemble learning models: light gradient boosting machine (LGBM), gradient boosting decision trees (GBDT), extreme gradient boosting (XGBoost), random forest (RF), and adaptive boosting (AdaBoost). The ensemble models were highly accurate, with r values of 0.82, 0.88, 0.64, and 0.86 for Voc, Jsc, FF, and PCE, respectively.

Previously, all works used molecular structure in conjunction with QSPR descriptors as input features, and multiple ML models were used to achieve varying accuracies. Some works performed a predictive model for OPVs without XAI such as in^12^, the authors developed the structure–property link using a supervised ML model to screen OPV materials quickly. Images, descriptors, and fingerprints are used as inputs for the predictive model that is based on the RF method to predict PCE. The dataset contains 1719 realistic OPV donor materials; the demonstrated prediction results achieve a correlation of r-value = 0.79.

The authors in^13^, utilized RF predictive model and quantum chemistry to develop novel OPV acceptor materials and to comprehend the structure–property relationships. The predictive performance of the trained predictive model is enhanced when molecules are encoded using an improved one-hot code. Consequently, 22 novel acceptors are removed from the virtual chemical space who’s anticipated PCE values are higher than 17%. Based on the observed patterns among the high-performing molecules, it can be inferred that a new material acceptor (Y6) derivatives featuring side chains of moderate length exhibited superior performance. Additional calculations in quantum chemistry demonstrated that the efficacy of OPV devices is primarily influenced by the electrostatic potential on the molecular surface and the frontier molecular orbital energy levels of the acceptor units. A series of prospective Y6 derivative candidates were eliminated and a guide to the logical design of high-performance OPV acceptors was presented. Based on dataset contains 1296 possible donor–acceptor combinations, the demonstrated results of this paper is 1.08 for Mean Absolute Error (MAE) and 0.66 for correlation coefficient.

In^14^, the authors proposed an automated methodology for predicting PCE of OPV at high speed. The first step in implementing the framework was to train it using the ensemble learning model LGBM, which can predict PCEs based on the physical and chemical properties of molecules using a small dataset of high-quality experimental data.

Then, using a graph neural network (GNN) to produce a dataset including a vast number of chemical structures and features, they created a deep learning model capable of accurately predicting molecular properties. They can predict PCEs based on chemical structure in a direct, fast, and accurate manner by integrating deep learning and ensemble learning. Based on a dataset of 440 small molecule/fullerene pairs and their accompanying PCEs, this paper’s results show r-value is 0.87 and Root Mean Square Error (RMSE) is 0.97.

Recently, Explainable Predictive Modeling has been used to predict and evaluate the performance of OPV in various research works. The majority of these works’ prediction models are based on ensemble learning integrated with SHAP to determine the influence that descriptors have, such as RF and GBDT, which were used in^15^ for different target parameters, RF for predicting the PCE and achieving a r value of 0.791, and GBDT for predicting both J_SC_ and V_OC_ and achieving r values of 0.842 and 0.862 respectively. In^16^, RF and Bagging were used to predict PCE, achieving R^2^ values of 0.892 and 0.887, respectively.

Additionally, XGBoost is applied in^17^ with SHAP’s integration to predict Voc as the target parameter with R^2^ = 0.7. Also, the authors in^18^ integrate SHAP with six ML models; RF and XGBoost achieved the best performance among them for predicting PCE. The authors in this work created an acceptor material for an OPV database using Y6 and its variations. To prepare the acceptor molecules for machine learning, they were split into three sections and encoded using an enhanced one-hot coding. Ten significant microscopic characteristics of OPVs and 240 data points of small molecule OPV systems were used to build the ML models that define the structure–property connections. The models indicated that the development of promising OPV materials is strongly reliant on molecular orbitals other than the highest and lowest, which are rarely considered during the OPV design phase. The results revealed that the electrostatic potential at the molecular surface and the frontier molecular orbital energy levels are mostly altered by the end acceptor unit of molecules, according to quantum chemistry calculations, which causes variations in photoelectric conversion efficiency. The introduced models based on RF and XGBoost were utilized to predict PCE with 240 small molecules of organic solar cells collected with 220 unique donor materials blended with two fullerene-based acceptors; the demonstrated results are 0.76 and 0.77 of R value for RF and XGBoost, respectively.

To the best of our knowledge, the proposed work is the first to develop an optimized model for the computational structural descriptors dataset, outperforming the five ensemble learning models that were primarily introduced and applied to this dataset in^7^. To highlight the key contribution of this work to the research gap, Table 1 below compares the proposed work to the related work based on input features, target parameters, and the methods used for machine learning, optimization, and XAI.

**Table 1:** Comparison of the proposed work against related work highlighting the research gap.

References	Input features	Target parameters	ML method	Optimization method	XAI method
Kim et al.^7^	Computational structural descriptors	Voc, Jsc, FF, and PCE	LGBM, GBDT, XGBoost, RF, and AdaBoost	–	–
Sun et al.^12^	Molecular structure + QSPR descriptors	PCE	RF	–	–
Zhang et al.^13^		PCE	RF	–	–
Wang et al.^14^		PCE	LGBM and GNN	–	–
Suthar et al.^15^		Voc, Jsc, and PCE	RF and GBDT	–	SHAP
Alwadai et al.^16^		PCE	RF and Bagging	–	SHAP
Lee^17^		Voc	XGBoost	–	SHAP
Abadi^18^		PCE	RF and XGBoost	–	SHAP
The proposed work	Computational structural descriptors	Voc, Jsc, FF, and PCE	Bagging	Bayesian Optimization	SHAP

## Basics and background

This section describes the OPV material screening and problem statement, followed by the proposed model’s construction methods, which include decision trees, ensemble bagging method, Bayesian optimization, and SHAP method.

### OPV screening and problem statement

Organic photovoltaic cells have a unique structure compared to conventional inorganic cells. The active layer is made of organic semiconductor materials, with conjugated polymers as electron donors and fullerene derivatives as electron acceptors. This layer is inserted between two electrodes and deposited on a transparent substrate, such as glass or polyethylene terephthalate. The anode is made of indium tin oxide, acting as a positive electrode for light flow and whole collection. With this structure, the light can pass via the transparent flexible substrate and the substrate utilized is glass or polyethylene terephthalate^19^. The photoactive layer is coated with a solvent-borne solution containing electron donors and acceptors. The cathode is made of aluminum, but silver or calcium can be used for electron collection. These techniques can manipulate molecular-level electron donors and acceptors, enhancing their potential in organic solar cells. Proper selection and pairing of materials is crucial for high efficiency and stability^20^. The electrical outputs of OPV cells, such as Voc, Jsc, and FF, are essentially what set the device’s efficiency and play a crucial role in assessing its overall performance. One of the most imperative characteristics of photovoltaic cells is their PCE, which designates how well the device transforms solar energy into electrical energy.

By measuring the open circuit voltage, short circuit current, and fill factor, the solar cell devices’ PCE can be measured. When a device is detached from a circuit, Voc detects the electrical potential difference between two terminals. The difference in energy between the acceptor’s and the donor’s is what regulates the maximum value of Voc. The maximum photocurrent density the apparatus is capable of constructing in a short circuit is denoted by Jsc. While FF sets the OPV device’s power conversion efficiency^21^.

### Decision tree algorithm

Decision Trees, a non-parametric supervised learning model, are used for regression and classification. There are three main kinds of nodes; parent nodes, internal nodes, and leaf nodes, in this tree-structured classifier. It is possible to further divide the root node—the original node that originally reflects the entire sample—into other nodes. A dataset’s attributes are represented by its interior nodes, and its decision-making processes are defined by its branching. The final result is shown on the leaf nodes. This method works quite well for handling decision-related issues. The most important aspects of decision tree algorithms are; they need less work to prepare the data, the data doesn’t need to be normalized, and having missing numbers in the dataset doesn’t change how the Decision Tree is built^22^.

The Decision Tree model makes it easy to get the answer. In this case, the data is said to be fragmented, and it often leads to overfitting. Pruning is usually used to eliminate stems that split on features that aren’t very important to simplify things and avoid overfitting. Purity statistics verify node divisions. If the tree has no output decision and a node is evenly split in half, it is 100% impure. Nodes are 100% pure when all input information matches one decision value. The highest purity improves accuracy. After splitting, the tree is trimmed to simplify.

The average is used as the final target value. Pruning reduces redundant or non-critical tree branches to predict output, as shown in Eq. 1^23^.

1 $$\Delta i\left(s. n\right)=i\left(n\right)- {F}_{l }i\left({n}_{l}\right)-{F}_{r}i\left({n}_{r}\right)$$

where $s$ represents a possible split at any node $n$, and ${F}_{l}$ and ${F}_{r}$ are the proportions of the left (${n}_{l}$) and right (${n}_{r}$) child nodes, respectively. In this case, $i(n)$ is a standard for impurity before splitting, and $\Delta i(s. n)$ is the final metric for reducing impurity after splitting $s$.

### Ensemble bagging method

An advanced machine learning method called ensemble learning involves training multiple independent models independently before combining them to improve predictions^24^. Bagging, which is also called bootstrap aggregation, is a type of ensemble learning that is often used to lower the variance in a noisy dataset, bagging can reduce the variance of an estimate. This offers a method for making a prediction more reliable^25^.

Let’s (y_1_, x_1_), …, (y_N_, x_N_) are the dependent variable is denoted by $yi \in R$ and the $p$ independent variables are $xi \in Rp$. $u = f(x)+u$ Denote the data generation process, where $E\left(u|x\right)= 0 and Var(u|x) = \sigma 2.$

An approximation function $\widehat{f}\left(x\right)$, such as linear regression, polynomial repression, or spherical regression, estimating the unknown conditional mean function of $y$ given $x. E(y|x) = f(x)$, by minimizing the L_2_ loss function.

2 $$Loss =\genfrac{}{}{0pt}{}{min}{\widehat{f}} \sum_{i=1}^{N}{({y}_{i}- \widehat{f}\left({x}_{i}\right))}^{2}$$

The overfitting risk can happen with this method if f̂(x) is a nonlinear function. For example, the ensemble error, which is represented by the mean square error of the ensemble, is denoted as Ensemble_MSE. The Ensemble_MSE is divided into bias and variance, as presented in Eq. 3:

3 $$\text{Ensemble}\_\text{MSE}=E{\left(y-\widehat{f}\left({x}_{i}\right) \right)}^{2} {=\left(E\widehat{f}\left({x}_{i}\right)-\widehat{f}\left({x}_{i}\right) \right)}^{2}+var \left(\widehat{f}\left({x}_{i}\right)\right)+var \left(u\right)={Bais}^{2}+variance+{\sigma }^{2}$$

The $\text{Ensemble}\_\text{MSE}$ consists of three components: bias, variance, and irreducible error variance $(\sigma 2 = Var(u))$. The bias and variance are governed by $\widehat{f}\left(x\right)$. A more sophisticated forecast $\widehat{f}\left(x\right)$ has a smaller bias. More complex $\widehat{f}\left(x\right)$ may have a higher variance. Minimizing the L_2_ loss function reduces bias, resulting in the ‘optimal’$\widehat{f}\left(x\right)$. Thus, $\widehat{f}\left(x\right)$ may not be resilient due to increased variance and errors.

This risks overfitting. Controlling the variance of $\widehat{f}\left(x\right)$ is necessary to tackle this issue. There are various ways to handle variation, such as integrating a regularization term or using random noise. Bagging is a strategy for controlling the variance of an estimated function $\widehat{f}\left(x\right)$ by averaging many models^26^.

### Bayesian optimization algorithm

Bayesian optimization is class of sequential optimization learning techniques for uncertain objective functions. BO is often recommended for finding the global optimum value since it can minimize the evaluation cost without relying on the function’s derivative. The goal of BO is to optimize the objective function by identifying the global optimum rather than settling for a local optimum. The surrogate function and the acquisition function are the two main elements of the Bayesian optimization approach, which is used to identify the optimal solution. A probabilistic distribution of objective functions is described in the first. As the probabilistic distribution is updated with results from previous iterations, the distribution’s mean and variance change dynamically. In previous studies, the Gaussian process (GP) was frequently used as a surrogate function. The acquisition function is a more streamlined and economical method in comparison to the surrogate function for identifying prospective spots that can be utilized in the next iteration to optimize the objective function^24^.

Different acquisition and surrogate functions may be assessed during the optimization procedure. The function that is being examined in this investigation is GP. This paper also examines and compares two acquisition functions that are frequently employed: expected improvement (EI) and probability improvement (PI). GP employs a stochastic method known as the Gaussian distribution. Depending on its mean and covariance, a multivariate normal distribution will be followed by certain random variables (x) in a Gaussian distribution. GP is a distribution across functions (f(x)) based on its mean and correlation values. GP creates a distribution that fits function values by using mean (μ) and covariance (k), rather than randomly selecting a value for each variable. Equation 4 introduces GP before the functions^25^:

4 $$p\left(f\right)=GP(f;\mu ;k)$$

where “k” stands for covariance and “$\mu$” for the mean value. By getting the results (observations) of each repetition, the distribution will be changed by using the following equation to condition the distribution, as show in Eq. 5.

5 $$p\left(f|O\right)=GP(f;{\mu }_{f|O};{k}_{f|O})$$

where “O” stands for the observation (x) and its function value ($f$) afterward.

The acquisition function seeks to update the surrogate function by balancing the utilization of existing knowledge with the exploration of new possibilities. Exploitation entails actively hunting for the best site despite higher degrees of uncertainty. The exploration entails sampling from the area designated in the exploitation phase to determine the site with the lowest mean value. Placing greater emphasis on either exploitation or exploration will not lead to the most effective option. The acquisition function aims to optimize the trade-off between exploiting current knowledge and exploring new possibilities in order to improve the performance of BO^27^. The commonly used acquisition functions, such as PI and EI, as previously stated. GP is used to apply the best-selected acquisition function and assess the BO on the RF hyper-parameter adjustment. The first function that is taken into consideration as an acquisition function is the PI. The following equation represents the PI function in its all aspects^28^:

6 $$PI=p\left(f\left(x\right)\le f\left({x}^{+} \right)\right)=\varphi \left(\frac{f\left({x}^{+} \right)- \mu \left(x\right)}{\sigma \left(x\right)}\right)$$

The probabilistic model created using a surrogate function has a mean and standard deviation of μ(x) and σ(x), a normal cumulative distribution function of φ(x), and an optimal objective function of f(${x}^{+}$). Equation 7 is used to determine the next possible point at which the process can be continued.

7 $$p{x}_{n+1={argmax}_{x\in X}PI(x)}$$

EI takes into account the optimal probability distribution supplied by the surrogate model in order to assess the expected improvement of f(x). The following is the EI equation:

8 $$EI\left(x\right)=\left\{\begin{array}{c}\left(f\left({x}^{+} \right)- \mu \left(x\right)\right)\varphi \left(Z\right)+\sigma \left(x\right)\varnothing \left(Z\right) if \sigma \left(x\right)>0 \\ \\ 0 if \sigma \left(x\right)=0\end{array}\right.$$

where φ(Z) is the probability distribution, $f\left({x}^{+}\right)$ is the best value of the objective function that has been observed thus far, and ∅(Z) is the standardized cumulative normal distribution. The Z’s equation is as follows:

9 $$Z=\frac{\mu \left(x\right)-f\left({x}^{+} \right)}{\sigma \left(x\right)}$$

Applying Eq. 10 will yield the next potential step in continuing the procedure.

10 $${x}_{n+1}={argmax}_{x\in X}EI(x)$$

### Shapley additive explanations (SHAP)

Explainable artificial intelligence is a well-established field that successfully explains and interprets predictions from complex algorithms. The goal is to help human specialists comprehend the underlying reasons for AI decisions. XAI is critical for causal knowledge because it provides responsible AI and transparent, verifiable machine learning in support of decisions^29^.

SHAP (Shapley Values) is an increasingly common method in XAI that provides a consistent and objective explanation of how each variable affects the model’s prediction output. The importance of variable $i$ is determined by examining how it affects the set $S$, which is a subset of variable indexes $S \subseteq \{1,..., p\}$. Its focus is on explanations for the model $f$ at a certain point ${x}^{*}$.

The model $f$ is based on a value function ${e}_{S}$, which can be described as the expected value of a conditional distribution where all variables in a subset of $S$ are subject to conditioning.

The weighted average of all potential subsets $S$ is used to calculate the contribution of variable $i$, indicated by $\varphi (i)$, as presented in the following equations^30^:

11 $$\varnothing \left(i\right)= \sum_{S\subseteq \{1,\dots ,p\}/\{i\}}\frac{\left|S\right|!\left(p-1-\left|S\right|\right)!}{p!}({e}_{s\cup \{i\}}-{e}_{s})$$

This equation equals

12 $$\varnothing \left(i\right)= \frac{1}{\left|\prod \right|}\sum_{\pi \in \prod }{e}_{before\left(\pi ,i\right)\bigcup \left\{i\right\}}-{e}_{before\left(\pi ,i\right)}$$

The set $\Pi$ comprises all orderings of p variables, and before (π, i) refers to the subset of variables that appear before variable i in the π order. A collection of values ${e}_{S}$, which shift from ${e}_{\varnothing }$ to f(x*), corresponds to each ordering. Averaging these contributions over all possible orderings yields SHAP. This method uses Shapley values to explain individual ML model predictions.

## The proposed organic photovoltaic prediction model

**Fig. 1:**
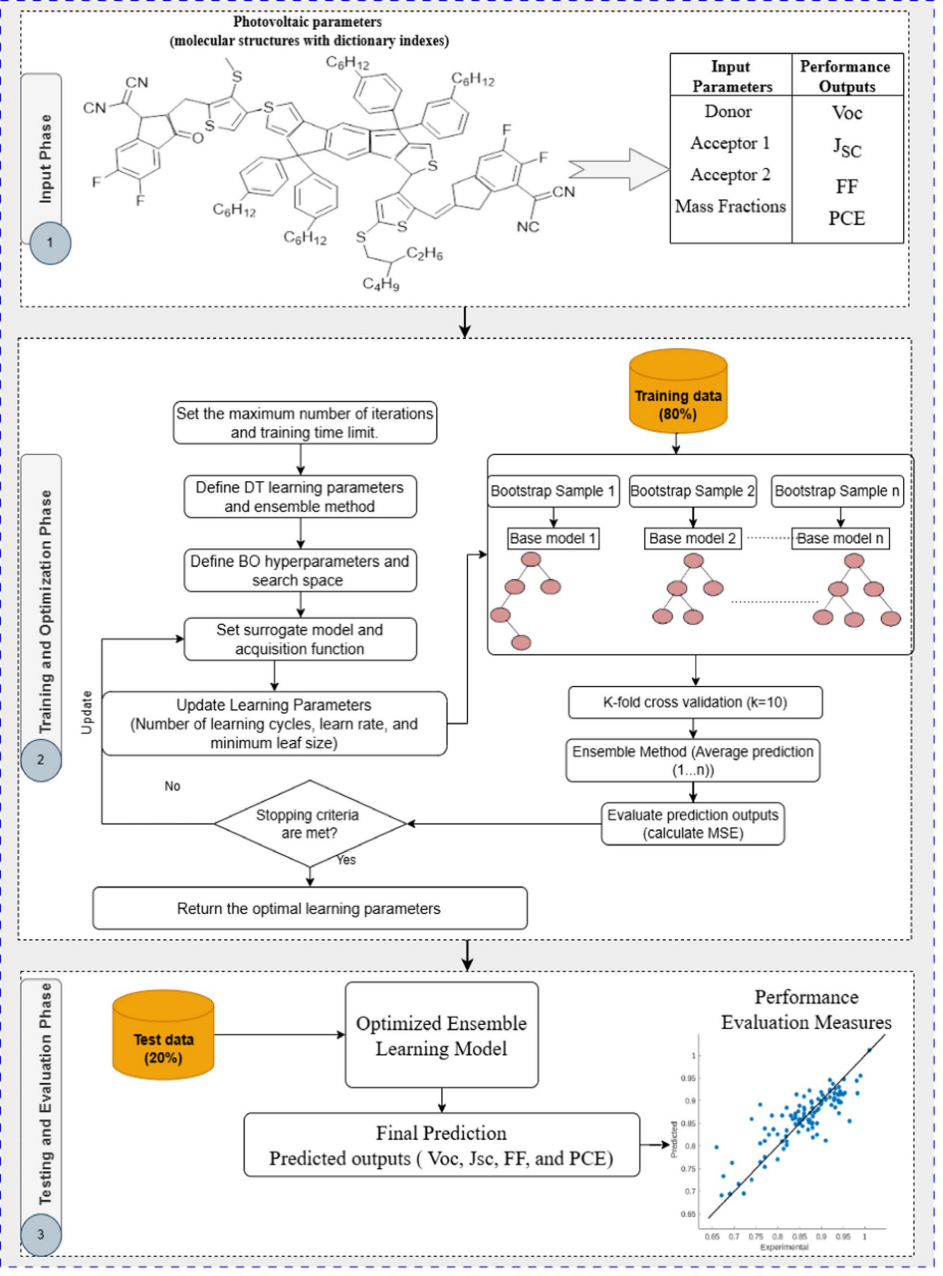
The Proposed Organic Photovoltaic Prediction Model Architecture.

BO-Bagging, the proposed optimized predictive model, accurately predicts four essential OPV parameters (Voc, Jsc, FF, and PCE) by Bayesian optimization and bootstrap aggregation trees. Ensemble learning is a sophisticated machine learning technique in which multiple independent models are trained separately and then combined to improve prediction accuracy. According to related research, ensemble learning is especially relevant in OPV prediction problems because of the data’s complex and high-dimensional structure. However, it may bring challenges such as overfitting, noise, and model selection. Bagging is a popular ensemble method that reduces variation and improves stability by training many models on different data subsets and then merging their results. Bagging is widely employed in a range of prediction situations due to its ability to improve model accuracy and robustness. While bagging enhances model robustness and accuracy, more effort is required to optimize hyperparameters or modify the model for specific targets, which has a significant impact on prediction accuracy and generalization performance. To fine-tune the prediction model and improve learning hyperparameters, Bayesian optimization is used. BO is a sequential search technique that is often substantially more efficient than grid or random search. It includes both exploration and exploitation. Algorithm 1 and Fig. 1 describe the proposed BO-Bagging model, which comprises several significant phases.

The dataset is split into 80% training and 20% testing. The training phase begins with setting a predefined number of iterations and a training time limit, defining bagging as an ensemble method, initializing learning hyperparameters for the bootstrap-aggregated decision tree ensemble (including minimum leaf size, number of learners, and number of predictors), specifying the BO search space, selecting a surrogate model and acquisition function for BO, and beginning with random initial learning parameters for the bootstrap aggregation tree.

The optimization process begins with the ensemble decision tree’s initialized hyperparameters, and the model’s prediction performance is assessed using the Mean Square Error (MSE) objective function until the stopping criteria are reached.

During the optimization phase, the learning parameters are improved, and cross-validation is used to assess the model’s performance and ensure that it can be successfully adapted to new data sets. Bayesian optimization creates a probability model of the objective function to efficiently identify the best learning hyperparameters for handling complex and nonlinear variable interactions. During the evaluation and output phase, the output-optimized learning model is applied to test data, and its performance is assessed using the evaluation criteria.

After obtaining the final prediction output and evaluation results for the test set. The explainable technique SHAP is applied to the optimized ensemble model to derive interpretability insights. Generate global summary graphs to visualize the influences of the selected optimal features for each target parameter. Also, generate dependence plots to assess the impact of each predictor on the predictive output.

**Algorithm 1:**
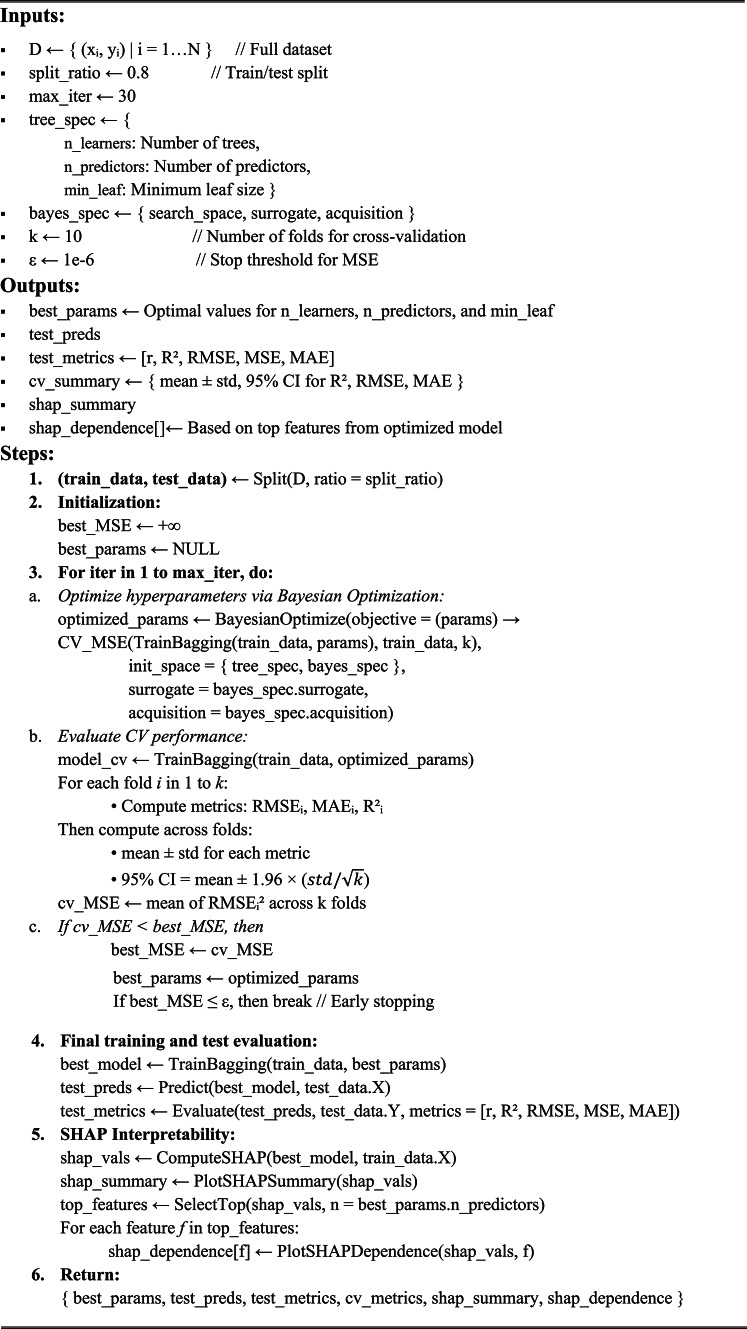
Proposed BO-Bagging Model with SHAP Interpretation

### Experimental analysis and results

The proposed experiment was conducted with the online MATLAB R2024a version, with statistical and machine learning toolboxes. The predictive performance of the proposed optimized model BO-Bagging is examined using a dataset that includes 755 samples, was separated into 80% and 20% for training and testing sets, respectively, with validation carried out using a K-fold cross-validation strategy with K = 10 folds. Table 2 shows the acquisition function settings, maximum iterations, and search ranges for optimizing hyperparameters in the Bayesian optimization configuration, which was used to select the highest-ranked features from a total of 48 features, with the training time limitation set to false.

**Table 2:** The optimization parameters and search ranges used in the proposed experiment.

Parameter	Value	Search range
Acquisition function	Expected Improvement per Second Plus	–
Max iterations	30	–
Optimized hyperparameters	Number of learners	10–500
	Minimum leaf size	1–300
	Number of predictors	1–48

### Dataset description

The dataset used in this paper includes 755 OPV samples with four target outputs of OPV performance metrics (PCE, Voc, Jsc, and FF). The predictors introduce OPV samples’ structural features, which are presented by 48 features that describe one donor and two acceptors and mass fractions. The structure of the dataset predictors and target responses is presented in Table 3.

**Table 3:** Predictors and target responses structure of the dataset.

48 predictors																			
Donor	BB1			BB2			BB3			BB4			BB5				BB6		
	SC1u	SC1l		SC2u	SC2l		SC3u		SC3l	SC4u	SC4l		SC5u	SC5l			SC6u		SC6l
Two acceptors	Acceptor1									Acceptor2									
	classA_1		EG1_1			pi1_1		Ring1_1		classA_2		EG1_2			pi1_2			Ring1_2	
	Ring2_1		Ring3_1			Ring4_1		Ring5_1		Ring2_2		Ring3_2			Ring4_2			Ring5_2	
	Ring6_1		Ring7_1			Ring8_1		Ring9_1		Ring6_2		Ring7_2			Ring8_2			Ring9_2	
	pi2_1					EG2_1				pi2_2					EG2_2				
Mass fractions	mass_fraction_1									mass_fraction_2									
4 target responses																			
Open-circuit voltage (V_OC_)			Short-circuit current density (J_SC_)						Fill factor (FF)							Power conversion efficiencies (PCEs)			

The predictors are structured as follows: Donor consists of six backbones (BBs), which are the basic structural frameworks of organic molecules or polymers. The backbones are structured from BB1 to BB6. Backbones include fragments side-chain (SC) that are functional groups or substituents attached to the backbone of an organic molecule or polymer. SC is categorized into upper (SCu) and lower (SCl) according to its position. The acceptor descriptor and can take values of 0, 1, or 2. “0” denotes a non-fullerene acceptor, “1” indicates a definite acceptor molecule called PC60BM, and “2” indicates another specific acceptor known as PC70BM. The structure includes samples that reflect several functional groups of chemical structures, with a focus on small molecule non-fullerene acceptor rings, small molecule non-fullerene acceptor π-bridges, polymer donor backbones and polymer donor side-chains. The main structural framework that affects the material’s overall stability and conductivity is the polymer donor backbone, while, the side-chains are important for shape and solubility, both of which are necessary for the effective processing of organic molecules. Non-fullerene acceptor components, such as rings and π-bridges can advance photovoltaic performance by contributing to charge transport and light absorption properties. The molecules can be represented as graphs for more complex modeling, especially with graph-based neural networks, where nodes stand in for atoms and edges for bonds, encapsulating the connections and interactions inside the molecular framework^7^. Samples of functional groups of molecular structures within the data set are presented in Table 4.

**Table 4:** Samples of functional groups of molecular structures. The functional group units belong to (a) polymer donor backbone, (b) polymer donor side-chain, (c) small molecule non-fullerene acceptor ring, (d) small molecule non-fullerene acceptor π-bridge, and (e) small molecule non-fullerene acceptor end-group dictionary.

(a)			(b)	
(c)			(d)	
(e)				

### Evaluation measures

More evaluation metrics are included to provide an in-depth evaluation of the model’s predictive accuracy, sensitivity, and ability to capture variability in target parameters, ensuring prediction dependability in the proposed model. The evaluation criteria utilized include Pearson’s correlation coefficient (r-value), coefficient of determination (R^2^), Mean Absolute Error (MAE), Mean Square Error (MSE), and Root Mean Square Error (RMSE). The r-value indicates the strength and direction of the linear relationship between predicted and actual values, while R^2^ represents the proportion of variance in the target variable that is predictable from independent variables, as shown in Eq. 13. Both measures, r-value and R^2^, have a range of 0 to 1. Higher values closer to 1 indicate a stronger correlation and better predictive performance^24^.

13 $${R}^{2}=1-\frac{\sum {\left({y}_{i}-{\widehat{y}}_{i}\right)}^{2}}{\sum {\left({y}_{i}-\overline{y }\right)}^{2}}$$

where ${y}_{i}$ is actual value, $\widehat{y}$ is predicted value, $\overline{y }$ is the mean value, and n is total number of samples.

MSE calculates the average squared difference between predicted and actual values, penalizing greater errors more heavily than MAE and thus being more sensitive to data outliers. RMSE is the square root of MSE, and it provides a more interpretable measure of error in the same units as the target variable^31^.

Regarding the complexity, the model has been assessed through five evaluation measures that are training time, inference time, and prediction speed, Floating Point Operations per Second (FLOPS), and model size that are used to evaluate the proposed model’s effectiveness^32^. All measurements were calculated separately based on the distinct input features for each target parameter to accurately determine the amounts of time and model size for each target parameter. First, the training time is calculated, which shows how long it takes to train the model on the dataset for each target. This includes the time needed to train the ensemble of decision trees and optimize with BO to find the optimal hyperparameters. The number of observations the model can predict per second for each target is represented by the prediction speed (obs/sec) measure. This measure calculates the inference time, which is the amount of time it takes a trained model to predict a single input sample for a single target^33^. Equation 12 presents the relationship between prediction speed ($${S}_{pred})$$ and inference time ($${T}_{inf})$$.

12 $${T}_{inf}=\frac{1}{{S}_{pred}}$$

The proposed model comprises a variety of floating-point operations, which are arithmetic calculations performed using floating-point numbers. These are the number of floating-point operations required to train and infer the ensemble of decision trees optimized using Bayesian Optimization. Several floating-point calculations are required during the optimization process to obtain the optimal hyperparameters, such as when the algorithm evaluates various configurations.

In addition, during the training process of the decision tree, a sequence of floating-point operations is used to split nodes, calculate information gain, and update parameters. Following that, the Bagging process, which generates repeated bootstrap samples from the training data, uses floating-point operations. So the computational complexity of a machine or deep learning model is calculated for each instance per second using the number of floating-point calculations required per second (FLOPS)^34^. In the proposed model, the FLOPS is the same for each target parameter since the same operations are performed by each process to predict each target parameter. Lastly, the model’s size evaluation measure indicates how much storage space is required to store it^35^. In the proposed model, the size will be determined by the amount of memory required in kilobytes to store Bayesian Optimization parameters as well as decision tree parameters such as split criteria, thresholds, and leaf values. Also, the number of decision trees in the bootstrap sampling procedure. The model size will differ for each target parameter based on the amount of input features. More features might result in more splits and nodes in the tree, which increases the model size.

### Results and analysis

The proposed model BO-Bagging is evaluated for its accuracy in predicting the OPV parameters. Table 5 displays the evaluation measures include R-value, R^2^, RMSE, MSE, and MAE metrics, which provide insight into the model’s prediction accuracy and performance in predicting Voc, Jsc, FF, and PCE. The model for Voc demonstrated a good correlation (r = 0.854) and accounted for 73% of the variance (R^2^ = 0.73). Similarly, Jsc shows a strong correlation (r = 0.917) and 84% variance (R^2^ = 0.84). FF had a moderate correlation (r = 0.734) and accounted for 54% of the variance’s cause (R^2^ = 0.54), but PCE had a high correlation (r = 0.866) and explained 75% of the variance (R^2^ = 0.75). The model’s predictions for Voc, Jsc, and PCE demonstrated low RMSE and MSE values, ranging from 0.04 to 0.16 and 0.002 to 2.56, respectively, indicating highly accurate results.

**Table 5:** The model performance in terms of the r value, R^2^, RMSE, and MAE based on experimental Voc, Jsc, FF, and PCE values, respectively.

Target parameters	Evaluation measures				
	*r-*value	R^2^	RMSE	MSE	MAE
*V*_OC_	0.854	0.73	0.0415	0.00172	0.0282
*J*_SC_	0.917	0.84	1.5988	2.5562	1.1954
FF	0.734	0.54	3.8919	15.147	2.9621
PCE	0.866	0.75	1.3310	1.7716	1.0019

While the FF model achieves larger errors, it remains acceptable in comparison to previous work. The lowest error reported by the RF model in related work is 4.46, while the worst error from AdaBoost is 4.59. This indicates that the proposed model has enhanced error rates by 0.57 to 0.7.

The MAE values showed the average absolute errors for each parameter, illustrating the model’s different levels of precision in predicting OPV parameters, with results ranging from 0.0282 to 2.9621, indicating high accuracy in the average absolute errors across all parameters.

To evaluate the stability and generalizability of the proposed model, the mean and standard deviation of the main performance metrics are computed across tenfold cross-validation, along with the 95% confidence intervals. Table 6 shows the mean ± standard deviation and the 95% confidence interval of MAE, RMSE, and R^2^ for each target parameter. The obtained results demonstrated consistent performance across tenfold cross-validation with low variance and a narrow confidence interval, indicating high confidence in the outputs, as well as statistical reliability for all measures for each parameter with strong generalization and consistency.

**Table 6:** Cross-validation results: mean ± standard deviation and 95% confidence intervals (CI) for each MAE, RMSE, and R^2^ over 10 folds.

Target parameters	Evaluation Measures	Mean ± Std	95% CI
*V*_OC_	MAE	0.0286 ± 0.0030	0.0286 ± 0.0019
	RMSE	0.0413 ± 0.0067	0.0413 ± 0.0041
	R^2^	0.6416 ± 0.1513	0.6416 ± 0.0938
*J*_SC_	MAE	1.3065 ± 0.1549	1.3065 ± 0.0960
	RMSE	1.7617 ± 0.2574	1.7617 ± 0.1595
	R^2^	0.8024 ± 0.0502	0.8024 ± 0.0311
FF	MAE	3.1294 ± 0.2837	3.1294 ± 0.1759
	RMSE	4.0105 ± 0.3323	4.0105 ± 0.2060
	R^2^	0.5059 ± 0.0471	0.5059 ± 0.0292
PCE	MAE	1.0157 ± 0.1467	1.0157 ± 0.0909
	RMSE	1.3357 ± 0.1859	1.3357 ± 0.1152
	R^2^	0.7512 ± 0.0844	0.7512 ± 0.0523

**Fig. 2:**
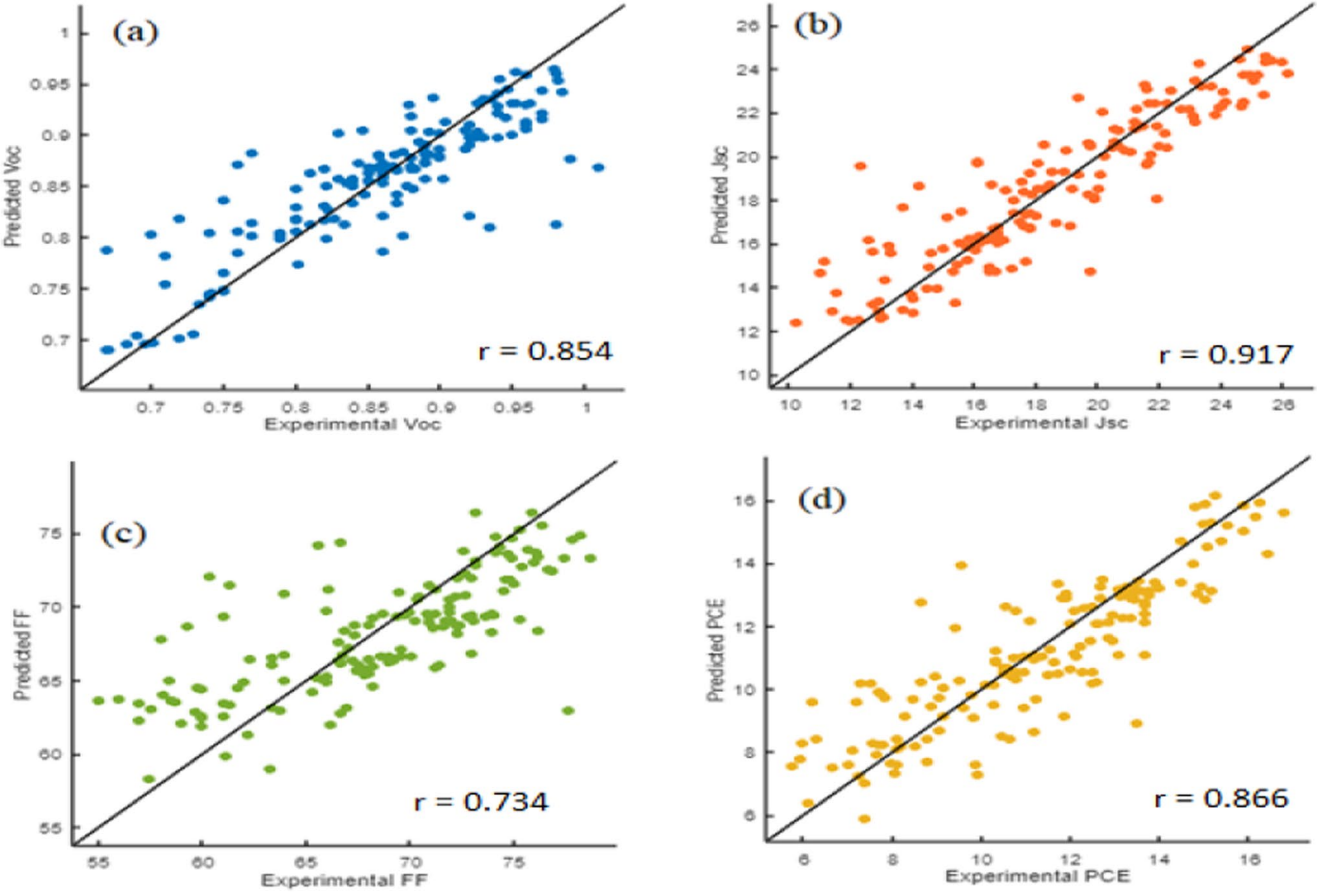
Scatter plots for predicted versus experimental values. Correlation between the experimental and predicted results of (**a**) Voc, (**b**) Jsc, (**c**) FF, and (**d**) PCE.

Scatter plots are used in Fig. 2 to demonstrate the correlation between experimental and predicted values for Voc, Jsc, FF, and PCE. Each subplot displays the relationship between experimental and predicted values, with the diagonal solid line representing a perfect positive correlation (r-value = 1.0), showing that the displayed outputs are consistent with the experimental data.

The correlation coefficients for Voc (r = 0.854), Jsc (r = 0.917), FF (r = 0.734), and PCE (r = 0.866) indicate how closely the experimental and predicted values for each parameter correspond.

The performance of the proposed BO-Bagging model is evaluated against five ensemble learning models introduced in^7^. Its performance is compared with LGBM, GBDT, XGBoost, RF, and AdaBoost based on r-value and RMSE metrics. As shown in Table 7, the proposed model had fewer prediction errors than the five ensemble learning models for all target parameters.

**Table 7:** Comparison with predication models in related work in terms of the *RMSE* for Voc, Jsc, FF, and PCE parameters, respectively.

Predictive model	Target parameters			
	*V*_OC_	*J*_SC_	FF	PCE
Proposed model (BO-Bagging)	**0.042**	**1.60**	**3.89**	**1.33**
LGBM	0.043	1.92	4.56	1.43
GBDT	0.043	1.93	4.52	1.41
XGB	**0.042**	2.08	4.56	1.44
RF	0.046	2.00	4.46	1.42
AdaB	0.045	1.98	4.59	1.42

Furthermore, as shown in Table 8, the proposed model outperformed the other five ensemble learning models in terms of the correlation between predicted and experimental values for all target parameters.

**Table 8:** Comparison with predication models in related work in terms of the *r* value for Voc, Jsc, FF, and PCE parameters, respectively.

Predictive model	Target parameters			
	*V*_OC_	*J*_SC_	FF	PCE
Proposed model (BO-Bagging)	**0.85**	**0.92**	***0.73***	**0.87**
LGBM	0.82	0.88	0.64	0.86
GBDT	0.81	0.88	0.62	0.86
XGB	0.82	0.86	0.62	0.85
RF	0.78	0.87	0.63	0.86
AdaB	0.79	0.87	0.62	0.86

The proposed model varies according to the selected predictors based on the optimization for each target response, resulting in variations in training time, inference time, prediction speed, and model size. The training time ranges from 105.2 to 296.9 s, the inference time ranges from 0.000216 to 0.000983 s, for prediction speed the number of predicted observed varies from 1017.47 to 4627.64 per second, and the model size ranges from 2352.52 to 19,336.63 KB. FLOPS has 108 floating-point arithmetic operations that represent the most critical operations in the training, optimization, and testing phases, such as bootstrap samples, tree constructions, aggregating the basic models to the final model, and hyper-parameters tuning during the optimization process. Table 9 presents the detailed results of the complexity evaluation measures.

**Table 9:** Training time, inference time, predication speed, FLOPS, and size of the proposed model.

Target parameters	Complexity measures				
	Training time (sec)	Inference time (sec)	Predication speed (obs/sec)	FLOPS	Model size (KB)
*V*_OC_	105.2	0.000216	4627.64	108	2352.52
*J*_SC_	124.29	0.000694	1440.96	108	8321.89
FF	296.9	0.000600	1667.48	108	12,950.57
PCE	204.5	0.000983	1017.47	108	19,336.63

**Fig. 3:**
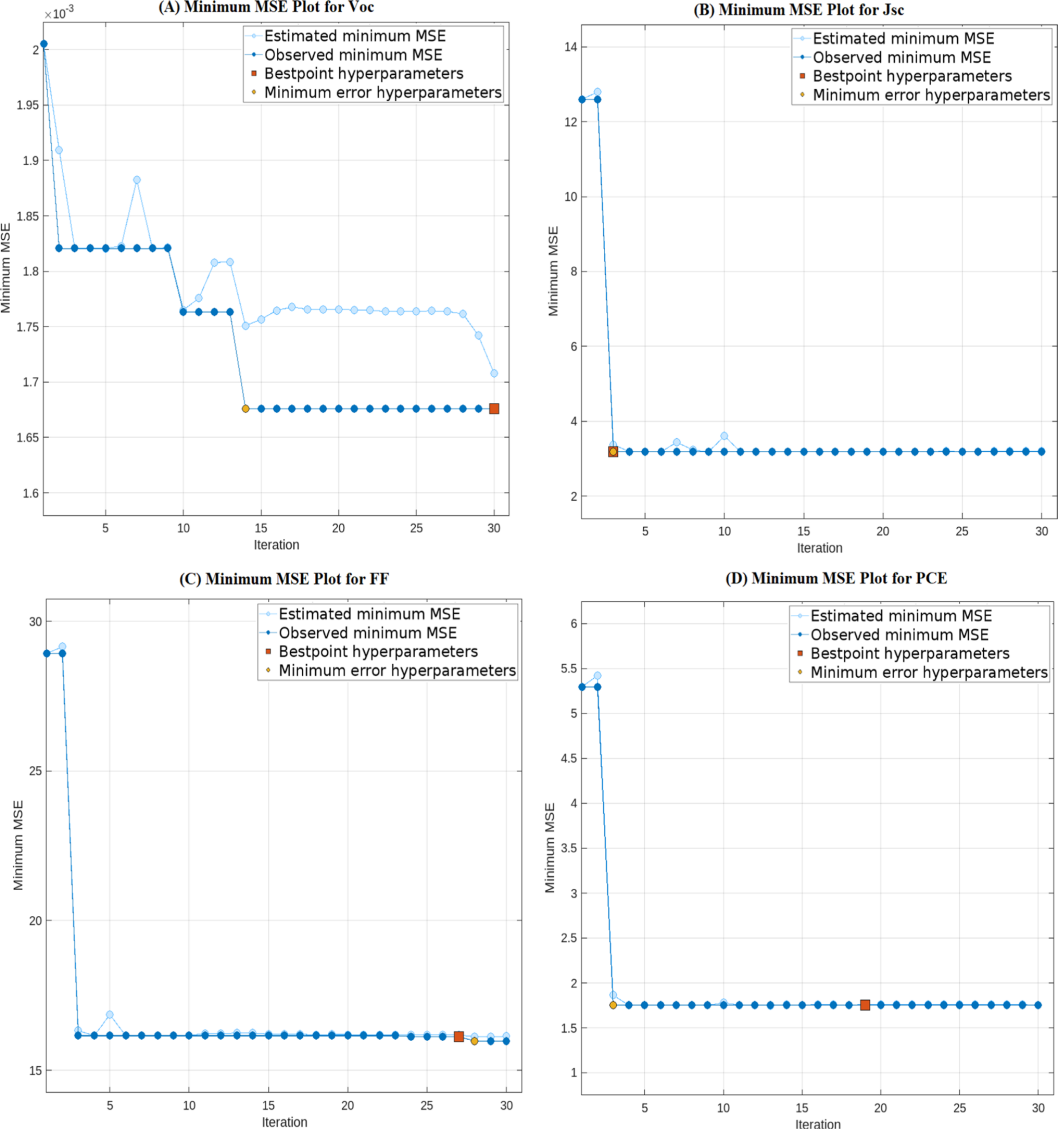
Min MSE versus the number of iterations for each target parameters.

The optimization process with the Bayesian optimization search-based process to tune and obtain the values of the optimal hyperparameters, is illustrated in Fig. 3. The plots show the observed and estimated MSE across 30 optimization iterations, displaying the minimum cross-validated MSE arising after determining optimal hyperparameters values for each target parameter, as shown in Table 10. The table shows the optimal number of learners, minimum leaf size, and number of predictors for each target parameter.

**Table 10:** Optimal Hyperparameters values using Bayesian Optimization.

Target parameter	Number of learners	Minimum leaf size	Number of predictors
Voc	387	2	15
Jsc	203	1	29
FF	327	1	19
PCE	492	1	35

All models exhibit stable performance after reaching their minimum observed errors within the search space, as shown by the optimization plots, which clearly show convergence and high stability.

**Fig. 4:**
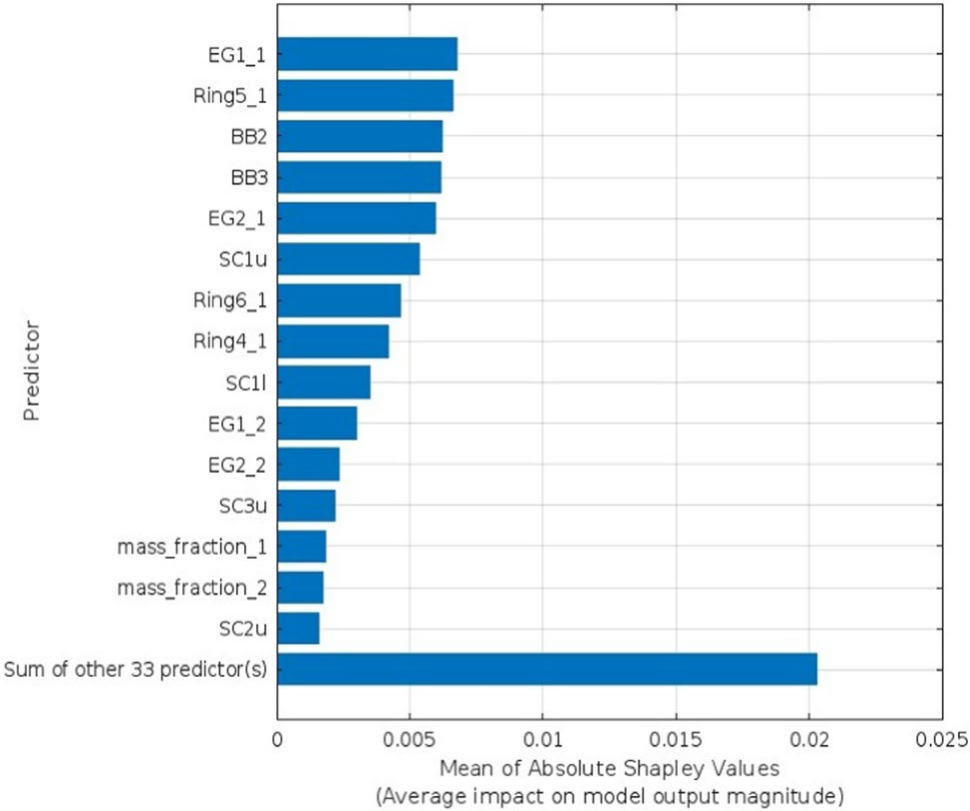
SHAP feature importance of the optimized predictors for Voc parameter.

**Fig. 5:**
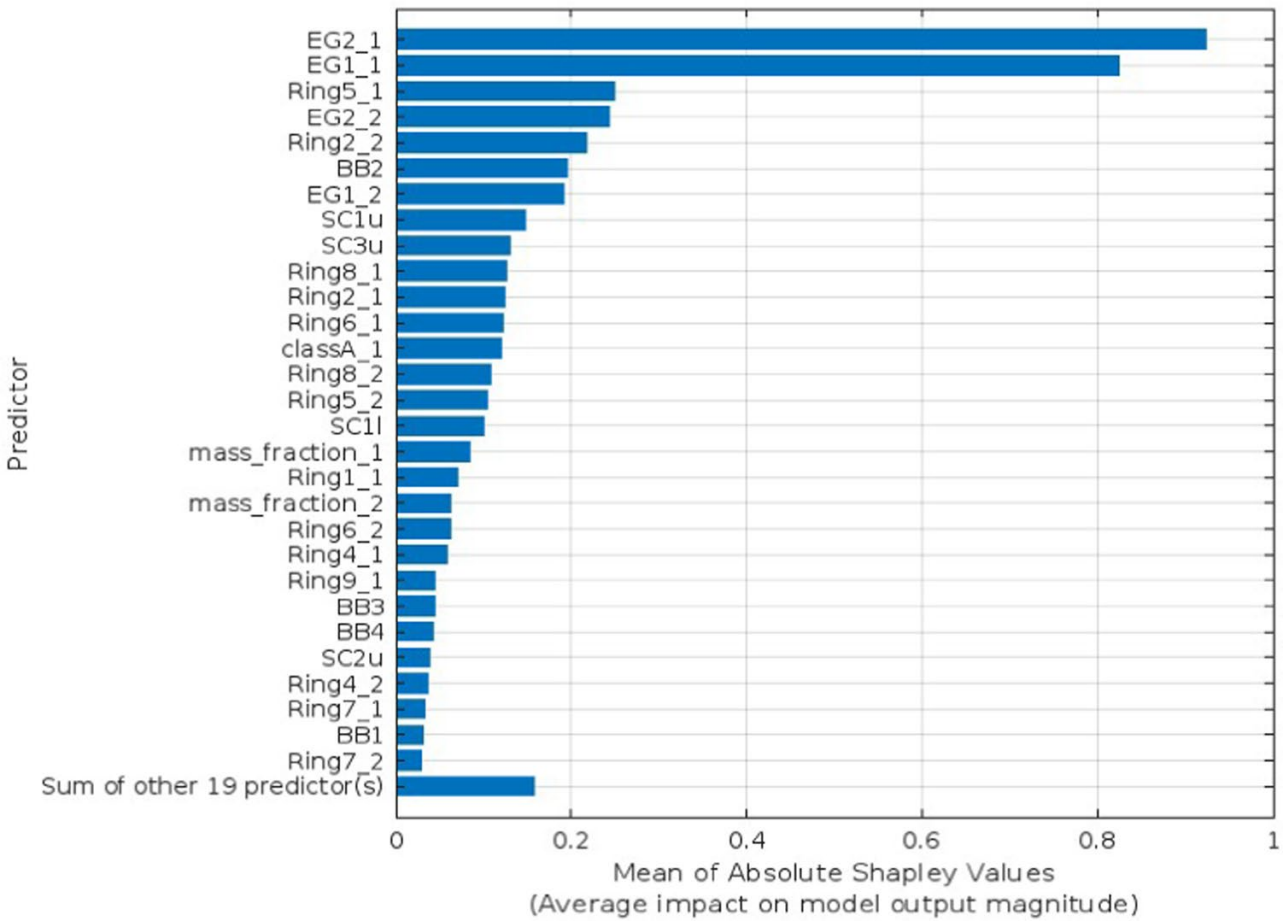
SHAP feature importance of the optimized predictors for Jsc parameter.

**Fig. 6:**
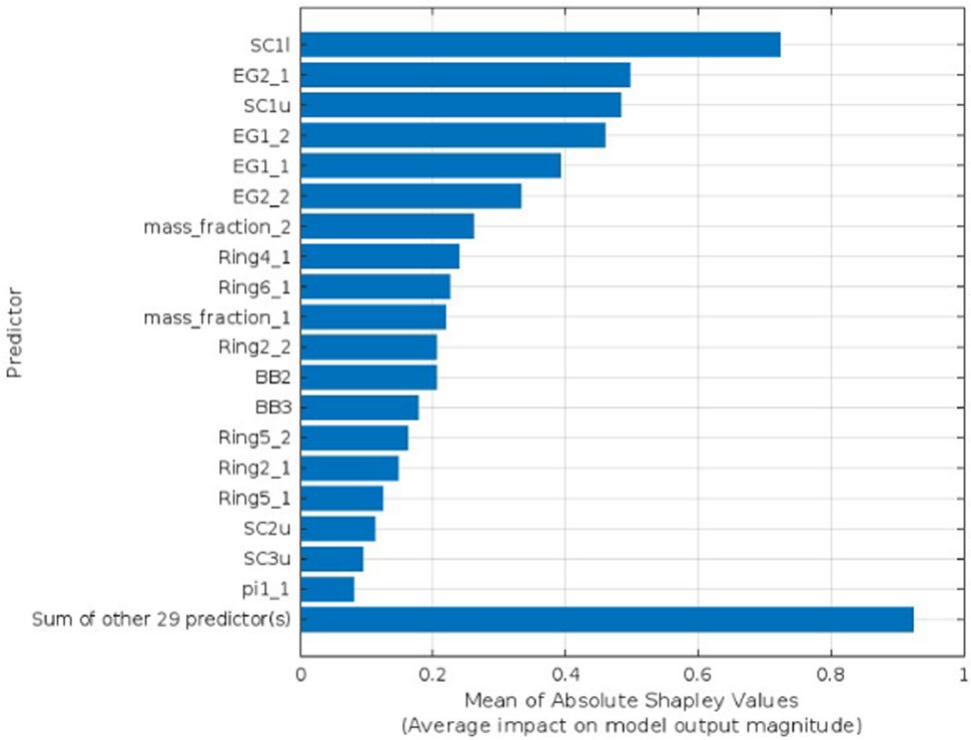
SHAP feature importance of the optimized predictors for FF parameter.

**Fig. 7:**
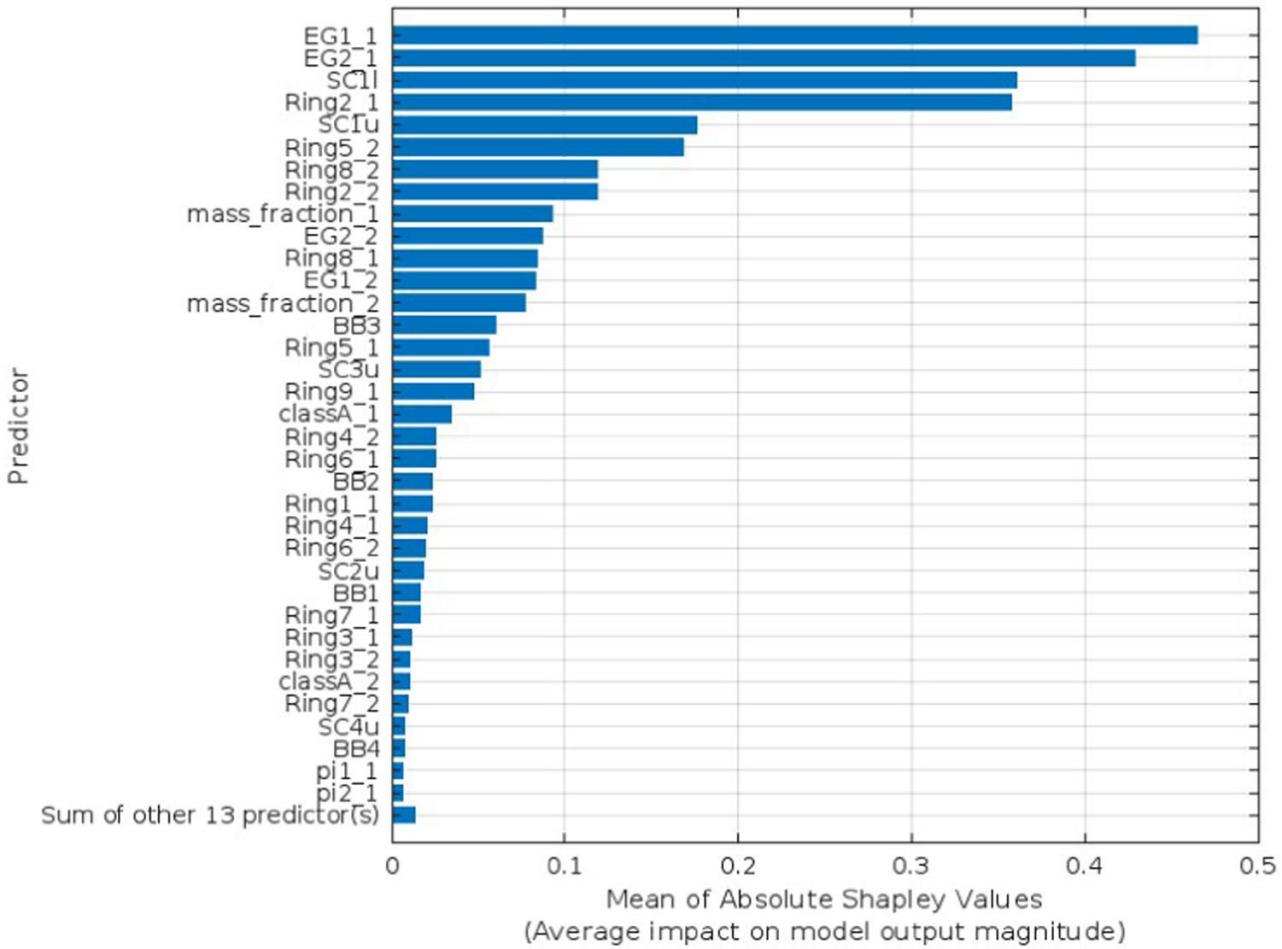
SHAP feature importance of the optimized predictors for PCE parameter.

Explainable AI is employed to interpret the selected predictors identified as the optimal number for each target parameter utilizing Bayesian Optimization. The SHAP as XAI technique interprets features’ influence on the prediction using bar lengths that represent the degree of impact a certain predictor has on a given target parameter, as shown in Figs. 4, 5, 6, and 7 for Voc, Jsc, FF, and PCE, respectively. Each bar’s length corresponds to the magnitude of the impact, with longer bars indicating greater impact. In each figure, show how the optimal number of predictors has a big influence on its target parameter.

The SHAP values validated the significance and impact of the selected optimal predictors for each target parameter through Bayesian optimization. Figure 4 shows the influence of the optimal 15 predictors, which means the other 33 predictors had no significant influence on the prediction. Similarly, in Fig. 5, FF has just 19 predictions out of 48. Unlike the Jsc and PCE, Figs. 6 and 7 retain the majority of predictors since they have a significant influence on the target parameter. In PCE, Bayesian optimization selects 35 predictors as the optimal number, which has been confirmed by the SHAP value in Fig. 7, which depicts the influence of each predictor until reaching the last one (Pi2-1).

**Fig. 8:**
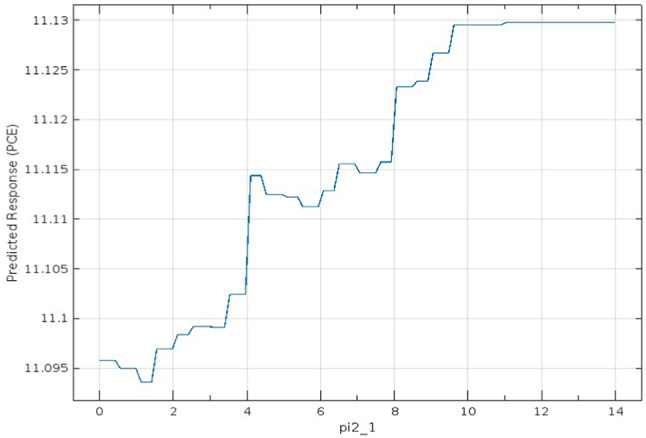
Partial dependence plot for the effect of feature pi2_1 on the predicted PCE value.

For evaluating the influence of this predictor on the PCE parameter, the dependence relation between Pi-2–1 and PCE is studied and presented in Fig. 8. The partial dependence plot in Fig. 8 shows the observed change and variation in PCE for pi2_1, reflecting a strong dependence, indicating that this feature has a significant influence on the model’s predictions.

**Fig. 9:**
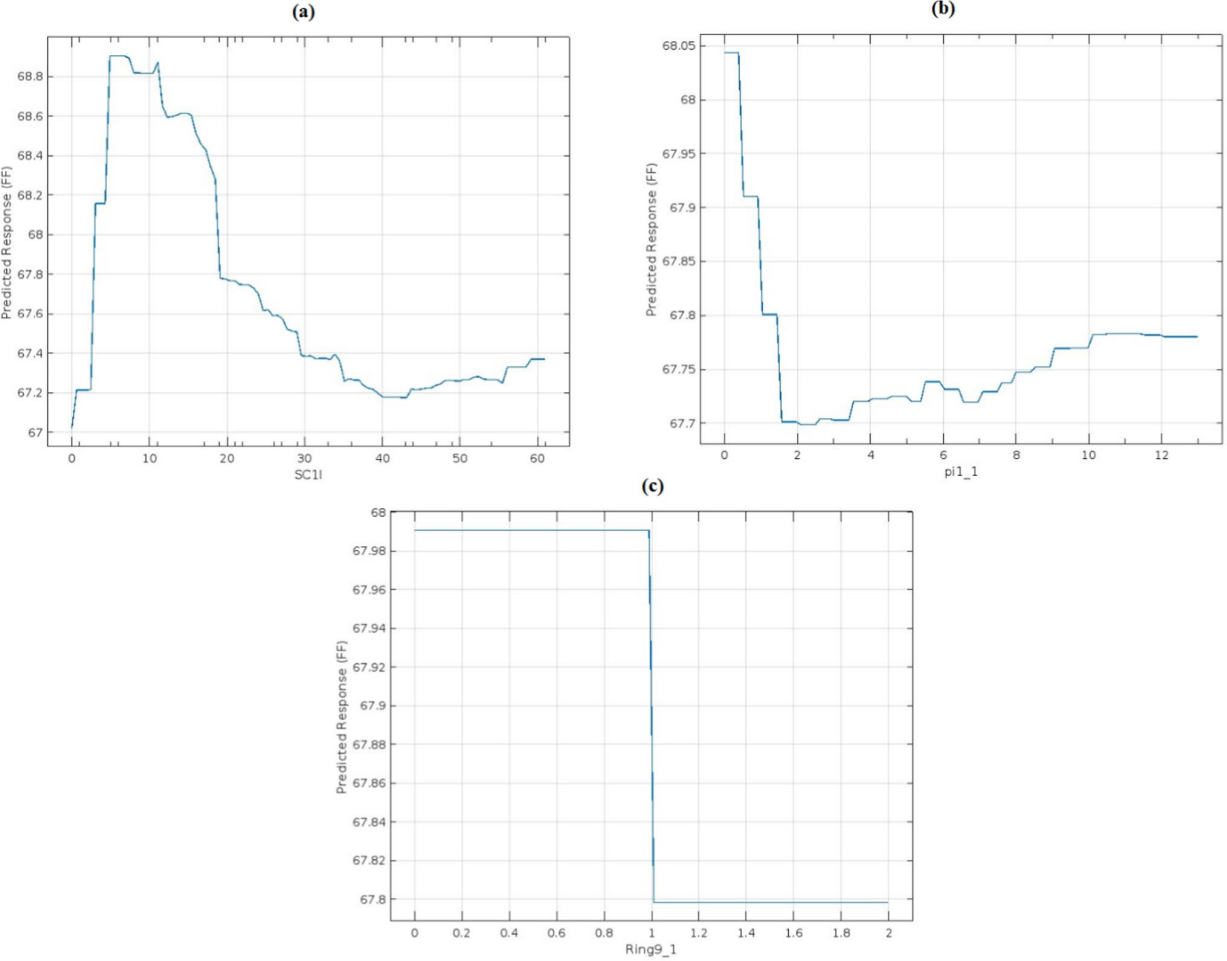
Partial dependence plot for the effect of three feature: (**a**) SCI1, (**b**) Pi1-1, and (**c**) Ring9-1 on the predicted FF value.

For another example, different dependence plots for the FF parameter with different features are illustrated in Fig. 9. The plots show three different influences on predicting FF by three features with varying degrees of influence on prediction. The first one is illustrated in Fig. 9a, which represents the plot dependence for the first optimal feature (SCI1), which has the biggest influence on the FF. The plot shows the big change and variation in FF with respect to SCI1, reflecting a strong dependence, indicating that this feature has a significant influence on the model’s predictions. The second is shown in Fig. 9b, which represents the dependence plot for the last feature (Pi1-1) in significance for FF prediction. The plot shows a strong dependence between FF and Pi1-1, confirming the importance of this feature among the selected optimal features during the optimization process. The third feature is the Ring 9-1, which is selected randomly from the other features that are not among the optimal features for FF. In Fig. 9c, the FF remains nearly constant across wide ranges of Ring 9-1, indicating that it has little influence on the prediction, validating the optimization results of eliminating it from the optimal hyperparameters.

## Conclusion and future work

Material selection and optimization techniques are critical for developing cost-effective, efficient fabrication methods and improving organic photovoltaic performance. The complexity of energy chemistry and the need for novel materials in solar cell efficiency and cost-effectiveness have led to challenges in establishing rules beyond empirical observations. Therefore, machine learning models are being developed to produce efficient predictive models that can effectively predict the photovoltaic parameters, which show potential for next-generation photovoltaic technology. This paper presents a novel hybrid-optimized predictive model, BO-Bagging, which demonstrates high accuracy and efficiency in predicting essential parameters for OPV cells. The proposed model holds promise for advancing the development of next-generation photovoltaic technology by enhancing material composition and power conversion efficiency. The proposed model integrates Bayesian optimization and Bootstrap aggregation decision tree techniques has shown superior performance compared to other ensemble learning models, with improved prediction accuracy and reduced complexity.

Finally, incorporating explainable AI into the proposed model and employing the SHAP feature importance analysis technique yields useful insights into the impact of particular features on prediction results. Future work could include more variables to increase the predictive model’s efficiency and reliability, such as temperature, illumination conditions, and device lifetime. Furthermore, certain factors can be considered to modify the dataset and improve the accuracy of predictive models, such as collecting data from a broader range of OPV cells with different types of solar cells, such as silicon-based solar cells (e.g., crystalline-silicon or amorphous silicon) or thin-film solar cells (e.g., cadmium telluride or copper indium gallium selenide). Another potential method for improving the collected datasets is data generation using deep learning techniques.

## Data Availability

The dataset used in this paper are benchmark datasets: Kim, gyu-hee, Novel Structural Feature-descriptor Platform for Machine Learning to Accelerate the Development of Organic Photovoltaics, Mendeley Data, V4, 2023. 10.17632/7wdzg9g2br.4
